# Uninterrupted embryonic growth leading to viviparous propagule formation in woody mangrove

**DOI:** 10.3389/fpls.2022.1061747

**Published:** 2023-01-04

**Authors:** Xiaoxuan Zhou, Yulin Weng, Wenyue Su, Congting Ye, Haidong Qu, Qingshun Quinn Li

**Affiliations:** ^1^ Key Laboratory of the Ministry of Education for Coastal and Wetland Ecosystems, College of the Environment and Ecology, Xiamen University, Xiamen, Fujian, China; ^2^ Biomedical Sciences, College of Dental Medicine, Western University of Health Sciences, Pomona, CA, United States

**Keywords:** vivipary, mangrove, embryo development, *LEC1*, *FUS3*, precocious germination

## Abstract

Vivipary is a rare sexual reproduction phenomenon where embryos germinate directly on the maternal plants. However, it is a common genetic event of woody mangroves in the Rhizophoraceae family. The ecological benefits of vivipary in mangroves include the nurturing of seedlings in harsh coastal and saline environments, but the genetic and molecular mechanisms of vivipary remain unclear. Here we investigate the viviparous embryo development and germination processes in mangrove *Kandelia obovata* by a transcriptomic approach. Many key biological pathways and functional genes were enriched in different tissues and stages, contributing to vivipary. Reduced production of abscisic acid set a non-dormant condition for the embryo to germinate directly. Genes involved in the metabolism of and response to other phytohormones (gibberellic acid, brassinosteroids, cytokinin, and auxin) are expressed precociously in the axis of non-vivipary stages, thus promoting the embryo to grow through the seed coat. Network analysis of these genes identified the central regulatory roles of *LEC1* and *FUS3*, which maintain embryo identity in Arabidopsis. Moreover, photosynthesis related pathways were significantly up-regulated in viviparous embryos, and substance transporter genes were highly expressed in the seed coat, suggesting a partial self-provision and maternal nursing. We conclude that the viviparous phenomenon is a combinatorial result of precocious loss of dormancy and enhanced germination potential during viviparous seed development. These results shed light on the relationship between seed development and germination, where the continual growth of the embryo replaces a biphasic phenomenon until a mature propagule is established.

## Introduction

Vivipary in flowering plants is defined as the precocious and continuous growth of the sexually produced offspring when still attached to the maternal parent (Tomlinson, 1986). Vivipary occurs in many families of plants. In some plants, this is genetic program driven, but in other plants it only occurs under certain environmental conditions. In crops, physiologically mature grains germinate in the ear or panicle, induced by high temperature and humidity shortly before harvest, termed pre-harvest sprouting (PHS) (Fang et al., 2008), which greatly reduces the grain quality and yield.

An extreme case of steady heritable vivipary as their only reproductive strategy occurs in mangrove Rhizophoraceae, in the genera of *Rhizophora*, *Bruguiera*, *Ceriops*, and *Kandelia* (Tomlinson, 1986). Because these mangroves live in a complex tropical intertidal habitat subject to high salinity, tidal waves, and unstable soil, their vivipary is considered an efficient strategy to protect the fragile propagules, permitting long distant sea dispersal and efficient seedling establishment (Tomlinson and Cox, 2000). Understanding the molecular and genetic mechanisms of mangrove vivipary will provide a valuable resource for expanding seed biology theory and is fundamental to guiding mangrove reforestation.

Morphological analysis of embryos in several viviparous mangrove species showed three developmental steps: cotyledon first growth, axis growth, and cotyledon second growth (Juncosa, 1982). Reduction of phytohormone abscisic acid (ABA) in viviparous mangrove species compared to non-mangrove relatives in the same family is considered a key to this phenomenon (Farnsworth and Farrant, 1998), and exogenous spraying of ABA inhibits the growth of viviparous fruit (Hong et al., 2018). The high water content of the reproductive organs, particularly embryos, ensures active metabolisms (Sussex, 1975). It was reported that the tandem duplicated GA biosynthetic gene ent-kaurene synthase (*KS*) and GA3β-hydroxylase (*GA3ox*) are candidate loci for vivipary in *Rhizophora apiculata* (Xu et al., 2017). Most recently, the mutation of the *DOG1* (*Delay Of Germination*) gene and associated transcriptional changes could be critical for vivipary development in *Kandelia obovata* (Qiao et al., 2020).

The most recent findings regarding vivipary molecular mechanisms in plants have come mainly from factitious mutants of Arabidopsis and cereals. In Arabidopsis, double mutants of *ABA1*, *ABI3*, *FUS3*, *LEC1*, and *LEC2* show precocious germination in siliques, such as *lec1-3 aba1-1*, *fus3-8 aba1-1*, *lec1-3 abi3-5*, and *lec2-1 abi3-5* (Raz et al., 2001). In rice, a series of potential pre-harvest sprouting genes were identified to encode the main enzymes in the carotenoid biosynthesis pathway that produces precursors of ABA, including ζ-carotene desaturase (*OsZDS*) and carotenoid isomerase (*OsCRTISO*) (Fang et al., 2008). Similarly, mutations of the *VP* genes, such as *VP1*, *VP5*, *VP7*, and *VP14*, blocked the biosynthesis of carotenoid or ABA signaling pathways were also sufficient to induce vivipary in maize (Durantini et al., 2008). However, the molecular process that generates naturally programmed vivipary in mangroves remains elusive.

It has been suggested that vivipary in mangroves should be regarded as a seed development process instead of an isolated ecological phenomenon (Tomlinson, 1986). Toward deciphering this genetic and molecular mystery, we performed a temporal-spatial seed developmental/germination transcriptome analysis in a typical viviparous species, *K. obovata*. Biological processes concerning developmental and germination were enriched in different stages and tissues, and a unique gene expression network was apparent during vivipary. This suggests that combinatorial effects of *LEC1*, *FUS3*, *ABI3* and hormonally related genes regulate the vivipary process.

## Materials and methods

### Sample collection

Flower and fruit samples of *K. obovata* were collected every 2 weeks between July and December in 2015. Samples for morphological observations were collected from Yuandang Lagoon (in Xiamen, Fujian, China) and fixed in formalin acetic acid (FAA) ethanol fixation solution at 4°C overnight, then dissected, and pictured with a Leica stereomicroscope. Flower and fruit samples for transcriptome analysis were obtained from the Zhangjiang Estuary Mangrove National Reserve (23°53’ 45” N–23°56’ N, 117°24’ 7” E–117°30’ E), Fujian, China, during July and December 2016. Samples were washed with ethanol, then put into RNAlater solution (Ambion Inc., USA) with 5% of β-mercaptoethanol, and stored at -20°C until use. Each sample has three biological replicates.

### RNA isolation

Tissues were dissected into seed coat and embryo components, and further separated into axis and cotyledon, before and after the axis protruding from the seed coat. RNA extractions were performed using MiniBEST Plant RNA Extraction Kit (TaKaRa).

### RNA-seq library construction and sequencing

RNA-seq library construction was based on Hunt’s protocol (Hunt, 2015) with modifications. Briefly, mRNA was enriched with Oligo d(T)_25_ Magnetic Beads (New England Biolabs), fragmented at 94°C for 3 min in 5× first strand buffer (Invitrogen), and reversely transcribed into cDNA by SMARTScribe (TaKaRa). Sample specific barcodes were incorporated into Illumina sequencing primers during PCR amplification by Phire II (Invitrogen), followed by 300–500 bp size selection using agarose gel electrophoresis, and purified by Zymoclean gel DNA recovery kit (Zymo Research). Library quality was assessed by High Sensitivity DNA Chips in the Agilent Bioanalyzer 2100. Qualified cDNA libraries were sequenced using the paired-end methods in the Illumina HiSeq 2500 platform at the College of Environment and Ecology, Xiamen University.

### Data processing and analysis

We used default parameters of Trimmomatic 0.36 (Bolger et al., 2014) to remove and filter low-quality sequencing reads, such as adaptor and barcodes, with reading lengths > 50 bp. FastQC analyses (Andrews, 2010) showed base scores of all processed sequences were higher than 20. Mapping quality was set at ≥ 20 when mapped to the reference genome by HISAT2 (Kim et al., 2019). Stringtie was applied to quantify the gene expression counts (Pertea et al., 2016). DESeq2 (Love et al., 2014) was used for gene expression normalization, principal component analysis (PCA), and pair-wise comparisons. R package WGCNA (Langfelder and Horvath, 2008) was applied to construct a gene co-expression network. Differential expressed genes (DEGs) were identified with |log2Fold change| > 1 and adjusted *p*-value < 0.05. GO enrichment analyses were performed by topGO (Alexa et al., 2019). Fisher’s exact test with a *p*-value threshold of 0.05 was used to filter KEGG enrichment results. Sample-specific genes were defined as genes with an expression level in a certain sample occupied over 80% of the total across all samples (≥ 20).

### RT-qPCR validation of RNA-seq data

Selected genes were validated by RT-qPCR of independent RNAs extracted from tissues of three independent biological replicates (few have only two independent biological replicates due a scarcity of samples). First-strand cDNA was synthesized using 5×All-In-One RT MasterMix (ABM, Canada), and qPCR was performed by CFX96TM Real-Time PCR Detection System (Bio-Rad, Inc.) using SYBR Green qPCR Kit (Roche, USA) and ChamQ SYBR^®^ qPCR Master Mix (Vazyme, China) according to manufacturer’s instructions. The relative quantification from three biological replications was normalized to the reference gene, *Ko-ACTIN*, and calculated by the 2^−ΔΔCt^ method. All primer sequences are shown in **Table S1**.

## Results

### Morphological changes during the viviparous developmental process

**Figure 1:**
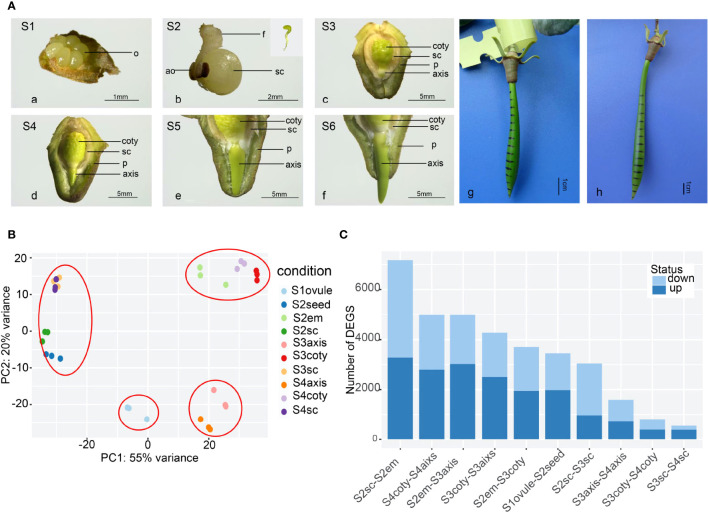
The reproductive process of viviparous and global gene-expression pattern in *K, obovata*. **(A)** The whole viviparous process from ovule to mature hypocotyl. (a) ovule stage (Stage1, S1); (b) enlarged growing seed (S2) and aborted ovules, the insert shows green and linear-shaped embryo; (c) developing seed about to germinate with the embryo enclosed in seed coat and pericarp (S3); (d) germinated seed whose axis protrudes out of the testa but within pericarp (S4); (e) elongating axis reached the edge of the pericarp (S5); (f) axis grows out of pericarp (S6); (g) with ink marked on hypocotyl in the field, 40d later, the upper zone in (h) show expansion while the rest remain the same. ao, aborted ovule; coty, cotyledon; f, funicle; em, embryo; o, ovule; p, pericarp; sc, seed coat. **(B)** Principal Component Analysis (PCA) of gene expression of 10 samples showing the three replicates of each sample were cluster well. **(C)** Differential Express Genes (DEGs) numbers in pair-wise comparisons of different tissues and stages from more to less showing tissue heterogeneity are more obvious than stage differences.

Vivipary in *K. obovata* was a successive and non-stop developmental process (**Figure 1A**). In mature flowers, six (occasionally four) semi-transparent ovules were arranged around the placenta (**Figure 1A** **-a**), and only one or occasionally two were fertilized and successfully grew into long liner-shaped embryos within 2 weeks (**Figure 1A** **-b**). There was no observable endosperm from this stage onward, indicating that it was degraded before the embryo was developed. After another month of growth, the embryo differentiated into cotyledon, and the axis was closely surrounded by the seed coat and pericarp (**Figure 1A** **-c**). The cotyledon expanded and occupied most of the seed cavity while small pits scattered around on the rough surface of the cotyledon. The axis was in a yellow-green oval shape while the shoot-hypocotyl-root structure started to appear. The seed coat covered the whole embryo tightly and presumably facilitated material transport and signal communication between the embryo and its maternal body. Later, the axis elongated and penetrated out of the seed coat (**Figure 1A** **-d**), indicating a transition from embryo development to seed germination, a critical point for vivipary. The axis was the functional tissue that facilitated this vivipary germination.

Constant elongation of the axis then penetrated out of the pericarp (**Figure 1A** **-e, f**). During this process, the embryo under the seed coat and pericarp was green-yellow. The color was lighter in the lower part of the seed coat and inner pericarp than in other parts, perhaps because of cell fibrillation. Field observation of marked hypocotyl growth showed that the shoot meristem in the upper zone was responsible for the hypocotyl elongation (**Figure 1** **A-g, h**), maintaining the hypocotyl growth until they were separated from the maternal tree and became an independent propagule.

### Transcriptome profiles across six tissues in four developmental stages

Transcriptome repertoires were obtained in four different developmental stages. They were unfertilized (Stage 1 or S1), fertilized (S2), non-vivipary (S3), and vivipary (S4) from ten representative samples (each with at least three replicates), namely ovule, seed, seed coat, embryo, axis, and cotyledons (**Figure 1A**). These samples were designated as S1ovule, S2seed, S2seed coat (or S2sc), S2embryo, S3axis, S3cotyledon (S3coty), S3seed coat (S3sc), S4axis, S4cotyledon (S4coty), and S4seed coat (S4sc), as brief sample names. We obtained ~387 Gb paired-end Illumina short-read data, and 732,979,784 high-quality reads after filtering and were used for the following transcriptome analysis. Alignment rates of these clean reads ranged from 79% to 92% after being mapped to the reference genome (Qiao et al., 2020) using HISAT2 (**Table S2**).

The high repeatability of three biological replicates of each sample was shown in the principal component analysis (PCA) based on gene expression profiles (**Figure 1B**). Furthermore, these samples could be divided into four groups according to the PCA: the seed coat (S3sc, S4sc, S2sc, and S2seed) cotyledon group (S3coty, S4coty and S2em), axis group (S3axis and S4axis), and S1ovule groups. This suggested that these samples were more consistent on the spatial scale than on the temporal scale. S2em grouped well with the other two cotyledon tissues, reflecting that the embryo was differentiated into cotyledon first in **Figure 1A**.

Pair-wise comparisons between the same tissues in different stages and vice versa identified many differentially expressed genes (DEGs) (**Figure 1C**), suggesting an intricate gene expression regulation profile during the viviparous process. The top DEG number was S2sc-S2em (7175); this high heterogeneity was probably due to these two representing the maternal tissues and the seed of a new generation. S3aixs-S4aixs (1591), S3coty-S4coty (810), and S3sc-S4sc (543) possessed lower DEGs number, suggesting that the gene expression dynamic between S3 and S4 was not as great as it appeared in morphology, although they finished the most important vivipary transition. There were 968 genes commonly highly expressed in S2em compared to S3em (both axis and cotyledon), and 666 up-regulated in S3. While comparing S3embryo with S4embryo, only 60 overlapped genes were higher in S3 and 113 in S4 (**Figure S1**). These numbers implied more gene regulation changes occurred in S2 to S3 than in S3 to S4.

### Gene-expression network analysis identifies biological processes and gene functions of viviparous embryonic tissue

**Figure 2:**
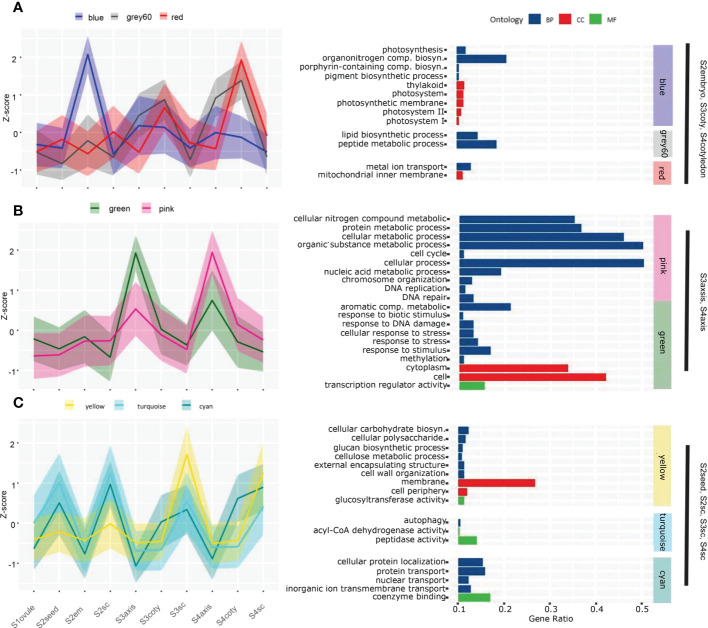
Gene co-expression pattern of each module in the course of the viviparous process. Gene expression levels in the different modules strongly correlated to certain tissues. A solid line represents the Z-score of total expression counts of all genes in the module; shadows delineate the SDs, and colors correspond to the module name. **(A)** The blue, grey60 and red modules are highly expressed in S2embryo, S3cotyledon and S4cotyledon. **(B)** The green and pink modules expressed higher in S3axis and S4axis than in other tissues. **(C)** Three modules that higher expressed in seed coats tissue; among them, the yellow module expressed maximum in S3sc and S4sc, while turquoise and cyan are higher in S2seed and S2sc. Enriched GO analysis of each module are given on the right-hand side panels, and the whole enrichment result was attached in **Table S5**.

Sequential viviparous development on a temporal-spatial scale could be revealed by their transcriptome dynamic. Therefore, we studied the overall expression network using Weighted Gene Co-expression Network Analysis (WGCNA). Thirty-three modules were obtained based on the pattern of 19290 expressed genes which account for 80.83% of total annotated genes (**Figure S2**). Each tissue had its specific or multiple modules with a robust correlation index (R) larger than 0.6. Gene ontology (GO) enrichment analysis of these highly correlated modules pinpointed the underlying biological processes that explain morphology changes in different tissues and phases, but some biological processes are not shown directly (**Figure 2**, **Table S3**).

S2embryo specifically correlated to the blue module (N=1894, R=0.82) that was enriched in photosynthesis events, including photosynthesis (GO:0015979), thylakoid (GO:0009579), photosystem (GO:0009521), porphyrin-containing compound biosynthetic process (GO:0006779), and pigment biosynthetic process (GO:0046148). A gene encoding light-harvesting complex II chlorophyll a/b binding protein was exclusively highly expressed in S2em than in other tissues (**Table S4**). This was in concordance with what we observed in **Figure 1A** that photosynthetic pigments could result in a green embryo appearance, indicating the early viviparous embryo were capable of conducting photosynthesis. These photosynthetic genes were also expressed higher in other embryonic tissues than the seed coat, although the correlation index was not as significant as in S2embyo (**Figure 2A**), suggesting that viviparous embryo could synthesize carbohydrates in all stages. Other substance accumulation pathways were also enriched in cotyledon tissues. Grey60 (N=1894, R=0.74), that was higher expressed in cotyledon tissues with a peak at S4coty, was enriched in pathways involved in lipid and peptide metabolisms. The red module (N=999, R=0.67) with a similar pattern was overrepresented in the mitochondrial inner membrane and metal ion transport. These two modules indicated an energy metabolism role in the cotyledon.

S3axis and S4aixs clustered in the pink (N= 1396, R=0.66) and green (N= 916, R=0.93) modules with higher expression of the green module in S3axis while the pink module expressed higher in the S4axis. These genes were mainly enriched in cellular metabolic and stress response pathways (**Figure 2B**). The former category comprised of cellular nitrogen compound metabolic process (GO:0034641), cytoplasm (GO:0005737), protein metabolic process (GO:0019538), cellular metabolic process (GO:0044237), cell cycle (GO:0007049), cell (GO:0005623), and DNA replication (GO:0006260). These pathways indicated rapid cell growth in axis elongation, which formed the foundation of germination. However, cellular aromatic compound metabolic process (GO:0006725), DNA repair (GO:0006281), response to stress (GO:0006950), response to stimulus (GO:0050896), and response to biotic stimulus (GO:0009607) play an important role in environmental stress tolerance. Specifically, these genes responded to UV, heat shock, osmotic stress, DNA mismatch repair, and hormone signals. They protected the axis, which developed into a propagule that finished the life cycle from the harsh coastal environment. In addition, the magenta (N=799, R=0.65) and orange (N=215, R=0.63) modules were exclusively related to the S3axis (**Figure S2**, **Table S3**), and genes were enriched in genetic material replication events, including nucleic acid metabolic process (GO:0090304), helicase activity (GO:0004386), RNA metabolic process (GO:0016070), DNA replication initiation (GO:0006270), and cell (GO:0005623). The S3axis was the most important tissue in breaking the surrounding boundary and accomplished vivipary germination by well preparation in genetic materials and cells.

Highly correlated modules showed their cell wall property and substance exchange capacity for maternal seed coats. The yellow module (N= 1707, R=0.96) is overrepresented in cellular carbohydrate biosynthetic process (GO:0034637), membrane (GO:0016020), glucan biosynthetic process (GO:0009250), cell periphery (GO:0071944), cellulose metabolic process (GO:0030243), external encapsulating structure organization (GO:0045229), and cell wall organization (GO:0071555), among others (**Figure 2C**). These processes were important for the expansion of the seed coat and facilitated testa loosening, which we observed in **Figure 1A**, thus providing room for embryo enlargement and protrusion. In this module, we found that 13 glucosyltransferase genes (sugar transporters) displayed maximum expression in S3sc, and showed energy provision of the maternal tissue (**Figure S3**). In the turquoise module (N= 2270, R=0.68), 70 genes were significantly enriched: in peptidase activity (GO:0008233), autophagy (GO:0006914), and acyl-CoA dehydrogenase activity (GO:0003995) pathways (**Figure 2C**). These genes act in the cleavage of peptide bonds, cellular degradation, and fatty acid β-oxidation. The overexpression of this protein hydrolysis and cell death genes might induce a loosening seed coat structure (**Figure S3**). Moreover, the cyan module (N= 451, R=0.66) is enriched in proton, protein, nuclear, inorganic ion, and nitrogen translocation pathways, implying the active substance interactions between maternal tissues and the embryo.

### Hormone-related gene networks identified central regulatory genes

The core regulation role of the hormone in plant growth has been extensively studied, especially abscisic acid (ABA) and gibberellin (GA). Crosstalk of various hormonal pathways may be more important than the effect of a single factor in deciding seed development and germination. To reveal the hormone regulation during mangrove vivipary, we identified 297 Arabidopsis phytohormone genes that functioned in biosynthesis, catabolism, signal transduction of ABA, GA, BR, cytokinin, auxin, and ethylene, and six homologs of transcription regulators (*FUS3*, *LEC1*, *LEC2*) were also acquired (**Table S5**). In total, 303 genes were re-extracted to construct the intricate gene expression network.

**Figure 3:**
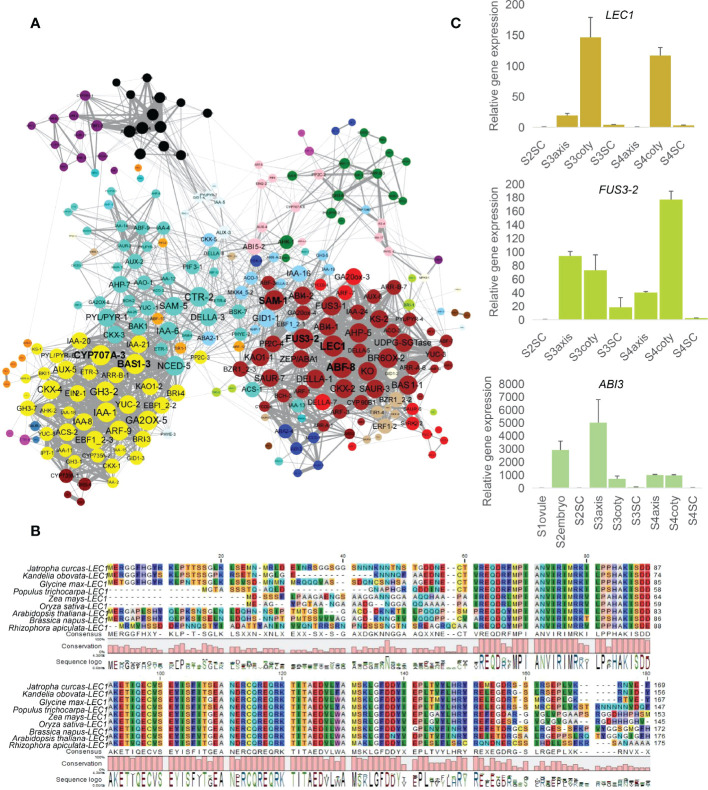
Network analysis of hormone-related genes and regulators. **(A)** Co-expression network of hormonally related genes whose nodes cutoff above p-value ≤ 0.01, color indicate the module attribute, the bigger circle size represents more connected genes. Yellow (lower-left), turquoise (upper-left), and brown (right) were bigger modules that survived under this filtered standard. Among them, six genes, namely *LEC1, ABF-8, SAM-1, FUS3-2* in the brown module and *CYP707A-3*, *BAS1-3* in the yellow module, correlated with more genes. **(B)** *LEC1* alignment in (*K*) *obovata* with eight dicot/monocot species showing conservative B domain from 62 to 149, numbers indicate amino acid position. **(C)** Expression levels of *LEC1, FUS3* and *ABI3* in RT-qPCR.

When performing gene network analysis, *LEC1*, *ABF-8*, *SAM-1*, *FUS3-2*, *CYP707A-3*, and *BAS1-3* were identified as hub genes in the central positions for their co-expression with another 52, 51, 49, 49, 48, 48 genes, respectively, filtered by the node cutoff of the p-value ≤ 0.01 (**Figure 3A**). *LEC1* and *FUS3* are primarily involved in the dominant embryogenesis process, especially cotyledon formation, and regulate seed dormancy (West et al., 1994; Wang and Perry, 2013). Orthologous *LEC1* amino acid sequences in *K. obovata* were aligned with other eight species and showed a conserved B domain position from 62 to 149 required for DNA binding and interaction with other CCAAT binding factors subunits (Lee et al., 2003) (**Figure 3B**). The phylogenetic tree among these monocot and dicot displayed that *LEC1* in *K. obovata* was close to that of another woody tree, *populous* (**Figure S4A**). *ABF-8* is an ABA-responsive factor, and *CYP707A-3* is an ABA catabolic gene (Okamoto et al., 2006). *SAM-1* involves ethylene biosynthesis (Gomez-Gomez, 1998), while *BAS1-3* is a major BR inactivating gene (Tanaka et al., 2005). Furthermore, *PP2C-3* was the only gene that connected the brown and yellow modules. It linked *BZR1_2-3*, *PP2C-4*, *SAM-1*, *ABF-3*, *KAO1-1*, *GA20ox-4*, *ABI4-2*, *FUS3-2*, and *SAUR-7* in the brown module with *ETR-3*, *IAA-21*, *IAA-20*, *EBF1_2-2*, *GH3-2*, and *BAS1-3* in the yellow module (**Figure S4B**). This array of genes might bridge the mutual communication between the cotyledon and seed coat through their co-expressed genes.

Consistent with the synergistic effect of *LEC1*, *FUS3*, and *ABI3* in embryo development (To et al., 2006), RT-qPCR showed their high expression in embryonic tissues (**Figure 3C**). We further explored the common downstream genes by this complex in *K. obovata*; genes response and/or biosynthesis of ABA, GA, auxin, and BR were co-regulated with the *LEC1-FUS3-ABI3* (**Table S6**). *ZEP/ABA1*, *ABA2-4*, and *BCH-3* are positively related to ABA content, while *PP2C-4* is a negative regulator of ABA responses (Raghavendra et al., 2010).

In this hormonal crosstalk, *LEC1—*as a key transcription factor through the regulation of downstream hormonal genes—might affect vivipary. Genetic and transgenic experiments have demonstrated that *LEC1* is a central regulator of seed development and acts at the highest level in the regulatory hierarchy controlling the maturation phase (Suzuki and McCarty, 2008; Jo et al., 2019). *Lec1* mutation led to vivipary in Arabidopsis and is required to specify cotyledon identity (Parcy et al., 1997; Lotan et al., 1998); conversely, *LEC1* expression in the endosperm can rescue *lec1* in the embryo (Song et al., 2021).

### Biological process and key genes during vivipary

**Figure 4:**
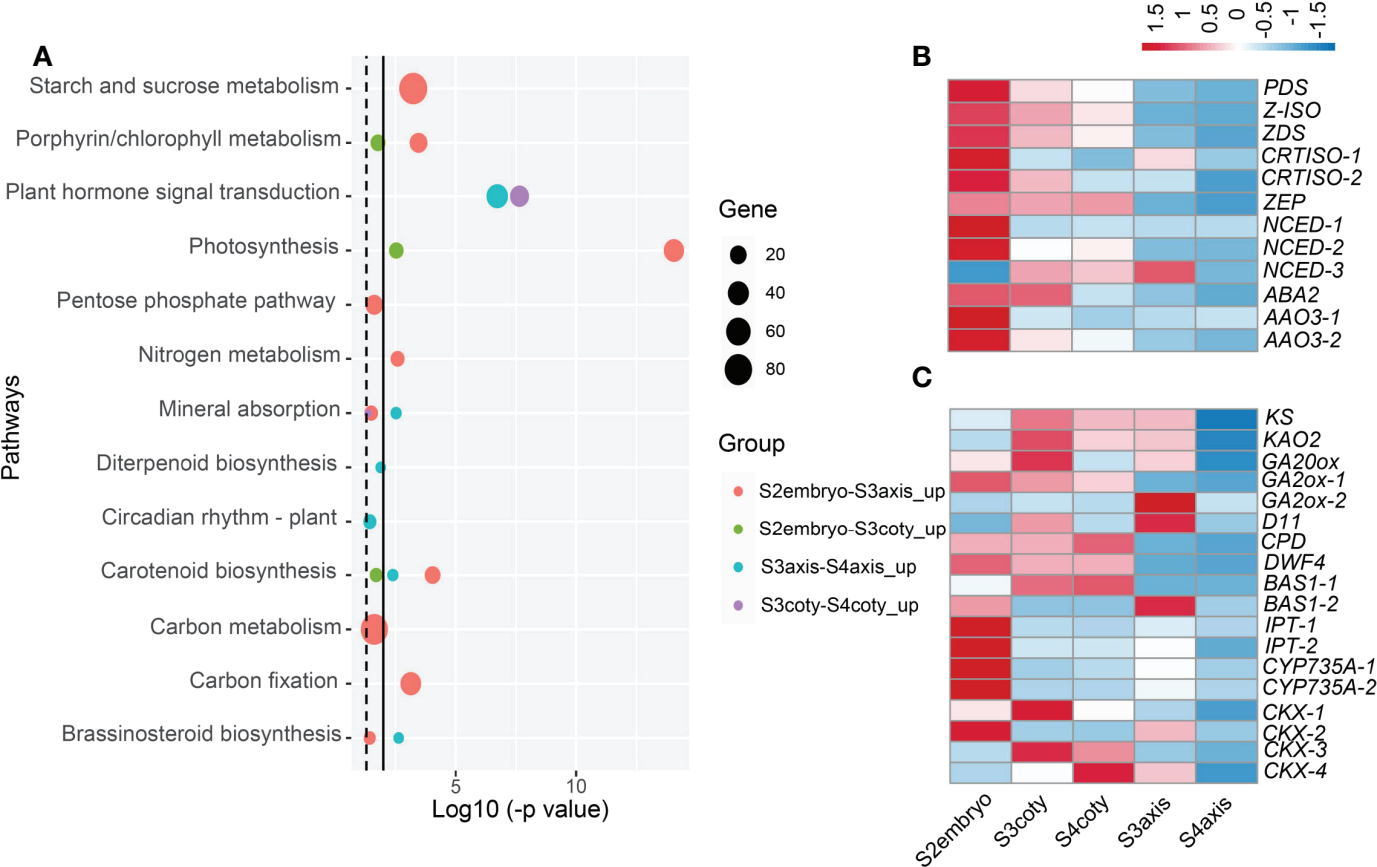
Candidate pathways and hormonal gene expression pattern during viviparous embryonic growth. **(A)** Enriched KEGG pathways compared embryonic tissues in adjacent stages; dotted line indicates p-value ≤ 0.05, while the solid line is ≤ 0.01. Circle colors represent different groups of genes, and circle sizes reflect gene numbers. **(B)** Expression heatmap of ABA biosynthesis genes, color means z-score, redder color means expression count with higher mean value while bluer is lower. Genes: *ABA2*, (*xanthoxin dehydrogenase*); *AAO*, (*abscisic-aldehyde oxidase*); *NCED*, (*9-cis-epoxycarotenoid dioxygenase*); *PDS*, (*15-cis-phytoene desaturase*); *ZEP*, (*zeaxanthin epoxidase*); *ZDS*, (*zeta-carotene desaturase*); **(C)** Expression pattern of GA, BR, cytokinin metabolism genes, *KAO2*, (*ent-kaurenoic acid oxidase*); *KS*, (*ent-kaurene synthase*); *GA20ox*, (*GA 20-oxidase*); *GA2ox*, (*GA 2-oxidase*); *D11*, (*CYP724B1*); *CPD*, (*CYP90A1*); *DWF4*, (*DWAF4*); *BAS*, (*CYP734A1*); *IPT*, (*isopentenyl transferase*); *CKX*, (*cytokinin dehydrogenase*).

In the viviparous process, the transition of embryonic growth to germination is accomplished seamlessly. To elucidate the molecular mechanism for such a transition, we investigated the development and germination-related pathways during vivipary through KEGG pathway analysis (**Figure 4A**, **Table S7**).

It was first noticed that photosynthesis (KEGG group ko00195) along with starch/sucrose metabolism (ko00500) and carbon fixation in photosynthetic organisms (ko00710) pathways were significantly higher in S2embryo compared with S3embryo, both in axis and cotyledon. Compared to S3axis, a respiration pathway, pentose phosphate that produces CO_2_ was enriched in S2embryo. In the porphyrin and chlorophyll metabolism pathways, we identified an *NYC1/NOL* (non-yellow coloring1/nyc1-like) gene encoding chlorophyll *b* reductase, which catabolizes chlorophyll breakdown (Nakajima et al., 2012). It showed a declined expression in the embryo after S2; in addition, this gene was unexpressed in S3coty and S4coty. This reduction of *NYC1/NOL* might result in less chlorophyll degradation, which helped keep the embryos in green.

Carotenoid biosynthesis, a pathway leading to ABA production (Ruiz-Sola and Rodríguez-Concepción, 2012), was significantly decreased in the viviparous embryo. It was a significantly higher expression in the S2embryo than S3embryo and higher in S3axis than S4axis, but there were no significant changes in S3 and S4 cotyledon tissues (**Figure 4A**). Among genes that participated in ABA biosynthesis, *PDS, Z-ISO*, *ZDS*, *CRTISO-1/-2*, *ZEP*, *NCED-1/-2*, *ABA2*, and *AAO3-1/3-2* reduced from S2 to S3 and showed the least expression in S4aixs, except *NCED-3*, which showed a different trend (**Figure 4B**). Therefore, ABA production declined from S2 to S3 and showed a minimum in S4. This pathway was up-regulated in cotyledon relative to the axis in both S3 and S4 (**Table S7**), suggesting that the cotyledon produces more ABA than the axis.

In the vivipary process, the signature of germination completion happens in the transition of S3 to S4. Mineral absorption and circadian rhythm pathways were enriched in S3. Further, three hormone biosynthesis pathways [diterpenoid biosynthesis (ko00904), brassinosteroid biosynthesis (ko00905), and zeatin biosynthesis (ko00908)] were overrepresented in S3axis than S4axis (**Figure 4A**). The gene expression patterns involved in these pathways are presented in **Figure 4C**. *KS, KAO2, GA20ox*, and *GA2ox* were in the GA metabolism pathway, while *CYP724B1/D11*, *CYP90B1/DWF4*, *CYP90A1/CPD*, and *CYP734A1/BAS1* were in BR metabolism, and *CYP735A*, *CKX*, and *IPT* in the CK metabolism steps. This result indicated that GA, BR, and CK positively regulated the viviparous process, given their well-known function in embryogenesis and seed germination. Moreover, plant hormone signal transduction (ko04075) was also overrepresented in S3 embryonic tissue than in S4 (**Figure 4A**). Eight genes annotated as *MYC2*, *jasmonate ZIM domain-containing protein*, *PIF4*, *ARR-A*, *ARR-B*, *BRI1*, *ARF*, and *IAA* that respond to JA, GA, BR, and auxin were commonly up-regulated in S3. Moreover, *PIF4* response to GA can stimulate hypocotyl elongation *via* interaction with BR and auxin (Mizuno, 2011; Eunkyoo et al., 2014). Thus, GA, BR, CK, and auxin act collectively in the growth of the axis.

As embryos grow older, the energy accumulation, cell growth, and stress response pathways are increasingly expressed (**Table S7**). Photosynthesis and sugar metabolism like starch and sucrose, fructose and mannose, galactose, respiration pathways, pentose phosphate, and glycolysis increased from S3 to S4. At the same time, certain growth-related events, including biosynthesis of amino acids, mineral absorption, circadian rhythms, and signal transduction pathways, such as light, calcium, cAMP, cGMP-PKG, and hormone, were also up-regulated. Particularly, DNA replication, mismatch repair, mRNA surveillance pathway, and cell cycle essential for rapid growth initiation were overrepresented in S3axis when compared to S2embryo. Lastly, phenolic compounds biosynthesis, mainly flavonoid and phenylpropanoid, were overrepresented in all up-regulated comparisons. Many secondary metabolites, like stilbenoid, diarylheptanoid, gingerol, ubiquinone and other terpenoid-quinone, and the indole alkaloid biosynthesis pathway were enriched in S3coty and S4axis compared to S2embryo and S3axis, respectively. Plant–pathogen interactions were also elevated in S4axis.

The above analyses indicated that the decrease of ABA and increase of GA, BR, CK, and auxin-related genes in S3 implied a well-prepared state for viviparous germination. Thus, we postulated that the “decision” of not going into a dormant state has been written in the transition from S2 to S3 but not in S3 to S4. The promoted energetic and stress-resistant substances in the embryos met the growth requirement under the complex habitat environment.

### Seed coat changes during vivipary

In addition to embryonic development, the seed coat is critical for controlling seed dormancy and growth. A lively and non-dormancy-imposed seed coat was presented in viviparous germination (**Figure 1A**). The molecular mechanism of seed coat changes in vivipary was revealed by its DEGs enrichment analysis.

**Figure 5:**
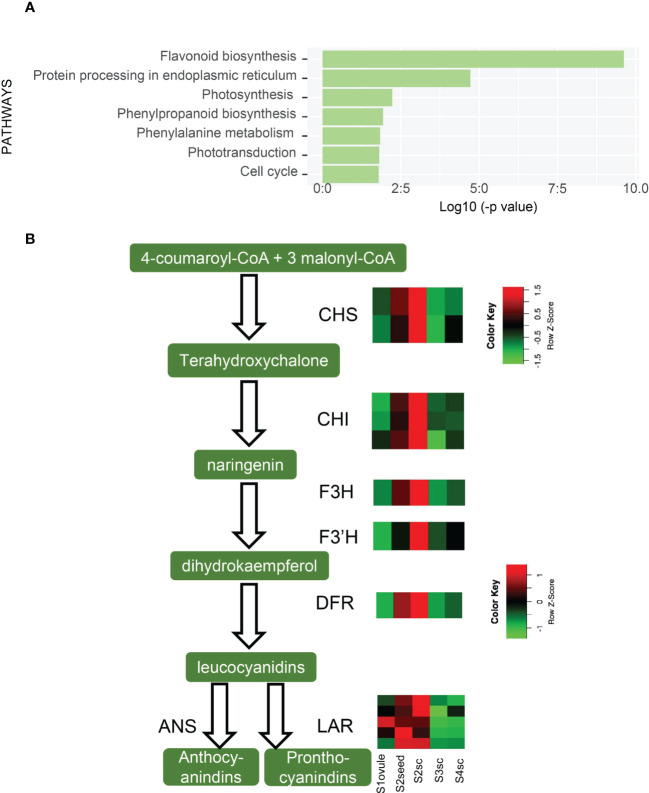
Up-regulated pathways of S3sc and seed coat-related gene expression. **(A)** Enriched KEGG pathways are higher expressed in S3sc compared to S2sc. **(B)** Flavonoid biosynthetic pathway and expression pattern of key genes involved. The expression levels are shown in heatmaps. The scale bars show the Z-score range. *CHS*, (*chalcone synthase*); *CHI*, (*chalcone-flavanone isomerase*); *F3H*, (*flavanone 3-hydroxylase*); *F3’H*, (*flavonoid 3’-hydroxylase*); *DFR*, (*dihydroflavonol 4-reductase*); *ANS*, (*anthocyanidin synthase*); *LAR*, (*leucoanthocyanidin reductase*).

In the gradual growth of the seed coat, the most significant change was the flavonoid biosynthesis pathway (ko00941), which decreased from S2 to S3 (**Figure 5A**).The flavonoid biosynthesis pathway produces proanthocyanidins (PAs) which compose the testa layer and induce seed coat-imposed dormancy (Debeaujon et al., 2003). Expression patterns of 15 DEGs functioned as *CHI*, *CHS*, *F3H*, *F3’H*, *DFR*, *LAR*, and *ANS* in flavonoid biosynthetic steps are shown in **Figure 5B**. This result showed that PAs increased from S1 and climax in S2, then decreased in S3 and remained not substantially changed in S4. We inferred that PAs declined from S2 to S3 to lighten the rigidity of the seed coat, thus making it easier for the embryo to breakthrough. Unlike being brownish and becoming dead tissue in the maturation period as in orthodox seeds, the *K. obovata* seed coat is retained in white and viable status throughout vivipary (**Figure 1A**). The reduction of PAs might contribute to the reduction of this vitality.

Along with the alleviation of PAs, S3sc possessed positive hormonal changes in zeatin and brassinosteroid biosynthesis pathways (**Table S7**). *IPT*, *CYP735A*, *CISZOG*, and *CKX* were up-regulated in S3 relative to S2sc, and *CYP724B1*, *DWF4*, and *BAS1* were also significantly higher in S3sc than S4sc. These gene expression patterns suggested the active role of CK and BR in the growth of seed coats. Meanwhile, hormone signal transduction genes were also significantly enriched in S3 sc. Therefore, seed coats might positively stimulate viviparous development resulting from PA reduction and hormone regulation.

The non-dormant state of the embryo and seed coat has been decided in the S2–S3 embryogenesis stage, while in S3–S4 are in preparation for germination that took effect ahead of S4, while the latter was more in an “after germination growth” state, a characteristic of vivipary.

## Discussion

Vivipary in mangroves has been recorded for over 100 years and appealed to ecologists for its adaptive role in the coastal environment. However, the detailed molecular mechanisms of vivipary were not studied until recently (Qiao et al., 2020; Feng et al., 2021). From a “seed biology” perspective, we investigated the transcriptome dynamics and pinpointed key regulatory circuits leading to viviparous development.

### Vivipary morphology is different from orthodox seeds

The essence of vivipary in mangroves lies in the offspring going through non-stop embryogenesis, germination, and growth while attached to the maternal tree. In our study, seed formation, embryogenesis, and germination were described as distinct stages (S1 to S4). Notably, S2 to S3 went through a cotyledon growth that fulfilled the seed cavity and the change of S3 to S4 mainly manifested in the axis protrusion, and the seed coat breakthrough marked the accomplishment of germination, which was the key transition from non-vivipary to vivipary. This growth pattern was consistent with other viviparous species (Juncosa, 1982). However, the absence of dormancy and germination in maternal are vastly different from the germination process of orthodox seeds like Arabidopsis, which go through a dormancy period and resume imbibition when seeds are permissive conditions free from the maternal plant (Rajjou et al., 2012). The long-lasting alive embryo and seed coat with developing/germinate capacity in vivipary were distinct characteristics from the quiescence embryo and dead testa in orthodox seeds.

### Functions of embryonic and maternal tissues during vivipary

Viviparous embryo and maternal seed coat play different roles in vivipary while also acting dependently. Photosynthesis-related pathways were enriched in S2embryo, S3, and S4cotyledons, indicating that the viviparous embryo of *K. obovata* can photosynthesize and assimilate with chloro-embryo during embryogenesis. Furthermore, glucosyltransferase and carbohydrate metabolism were found in the seed coat when the embryo exhibited significant changes in the respiration pathway. It was reported that the main carbon source for seed photosynthesis comes from the mother plant, and CO_2_ is released during seed respiration (Smolikova and Medvedev, 2016). Therefore, the viviparous embryo depends on the sucrose provision from maternal tissues and fixed CO_2_ on its own.

Photosynthesis augments the processing of carbohydrates derived from maternal sources during early development and also supports the differentiation of viviparous hypocotyl, which is an adaptive trait (Smith and Snedaker, 2000). Accordingly, our results showed that these photosynthetic genes are expressed as early as in S2; this finding extends the old stereotype that photosynthesis occurs until hypocotyl exposure to light and air (Tomlinson, 1986). Photosynthetic gene expression in S2 may be also related to LEC1, where the impact of LEC1 on photosynthesis have been documented in both monocotyledon and dicotyledon plants (Pelletier et al., 2017; Guo et al., 2021). The chlorophyll metabolism pathway, another factor involved in photosynthesis, is reduced from S2 to S3. This process might relate to a critical chlorophyll degradation gene *NYC*. As previously reported, the disintegration of photosynthetic apparatus and chlorophyll breakdown are prerequisites for dormancy (Galina et al., 2017). The failure of chlorophyll degradation in viviparous embryos contrasts with what happened in Arabidopsis seed, where degradation of their chlorophyll occurs during maturation. Therefore, the persistent green color in the viviparous embryo is also important in maintaining a non-dormancy embryo state.

Active axis cell growth and loosened seed coat facilitated axis protrusion out of the seed cavity. The enriched cellular metabolic pathways in preparing cytoplasm, cell and genetic materials constituted the driving force of S3aixs and S4axis. This is reasonable because hypocotyl elongation was the second growth phase in vivipary and was more rapid than the first cotyledon growth (Juncosa, 1982). The seed coat exhibited a cell wall and structure reorganization property, as shown in the gene expression profile, which made its enlargement easier to accommodate the increasing size of the embryo.

### *LEC1* and *FUS3* regulate viviparous embryo identity and germination

Co-expression network analysis identified the master regulator’s role of *LEC1* and *FUS3* during vivipary *via* interacting with other hormonal-related genes. In vivipary, *LEC1* and *FUS3* are prevalently expressed in the embryo, mainly in the cotyledon, even after germination (**Figure 3C**). It was noticed that viviparous embryos exhibited a long period of embryonic status, especially cotyledon maintained its characteristic till hypocotyl abscission (**Figure 1A**). However, in Arabidopsis, *LEC1* and *FUS3* are expressed from the globular stage and then shut down at the late seed maturation (Holdsworth et al., 1999). *LEC1* and *FUS3* are indispensable for suspensor and cotyledon identity (West et al., 1994; Wang and Perry, 2013). Overexpression of *LEC1* transgenic plant shows somatic embryogenesis in vegetative growth, strongly indicating the embryonic maintenance function of *LEC1* (Lotan et al., 1998), as further elucidated by a recent publication (Song et al., 2021). It has been found that endosperm expressing LEC1 is essential for embryo maturation (Song et al., 2021). However, there is no apparent endosperm after S2 in *K. obovata* embryos. This could provide another clue that viviparous seeds do not mature/dormant as orthodox seeds. Therefore, we inferred that the viviparous embryonic maintenance was determined by the action of *LEC1* and *FUS3*.

*LEC1* and *FUS3* also interacted with GA, auxin, and BR-related genes during vivipary. GA biosynthesis genes (*KO*, *KAO*, *KS*, *GA20ox*, and *DELLAs*) were co-expressed with *LEC1* and *FUS3* and correlated with auxin and BR biosynthesis genes, *YUC*, *IAAs*, and *BR6ox* in *K. obovata.* It was reported that *FUS3* represses GA biosynthesis and increases ABA in seed maturation (Gazzarrini et al., 2004). However, a study also showed that *LEC1* interacts with *DELLA in vivo*, and the increase of GA will release the suppressing role of *DELLA* to *LEC1*; in reverse, promoting auxin accumulation facilitates embryo development in late embryogenesis (Hu et al., 2018). *LEC1* targeted auxin biosynthesis and response genes, *YUC10*, *IAA16*, and *IAA19*, and the BR biosynthesis gene *DWF4* to promote hypocotyl elongation (Junker et al., 2012). In addition, the *LEC1* overexpressing seedlings showed hypocotyl elongation and hook formation similar to etiolation (Junker et al., 2012); this phenotype hinted the function of *LEC1* in viviparous seedling elongation. Therefore, in *K. obovata*, *LEC1* and *FUS3* may also stimulate axis protrusion.

*LEC1*, *FUS3*, and *ABI3* were tightly co-expressed in *K. obovata*, as seen in other plants, indicating the conservative function of this complex (To et al., 2006). Although *LEC2* was not in this group because of its absence of expression, its function might be compensated by *LEC1* and *FUS3*. Gene mutation of *LEC1/FUS3* and/or genes related to ABA biosynthesis/response such as *lec1-1/abi3*, *vps* performed precocious germination (Raz et al., 2001; Durantini et al., 2008; Fang et al., 2008). However, we found no mutations in these genes, perhaps because they have an indispensable function in development and stress responses. Instead, the viviparous embryo is more like a “*LEC1*-overexpression” line simultaneously showing embryonic and vegetative growth. *LEC1* was also co-expressed with other ABA response genes, including ABF. It was reported that *LEC1* induces the seed storage protein in seed maturation by directly targeting ABA-responsive elements (ABREs) in their promoters (Kagaya et al., 2005). Since there is no desiccation period in vivipary, even *LEC1* co-expressed with ABF, whether or how *LEC1* regulates protein and lipid storage *via* ABA is not clear.

Taken together, the above results show that *LEC1* and *FUS3* functioned in sustaining the embryogenesis state and perhaps stimulated viviparous germination through an interactome with ABA, GA, BR, and auxin.

### Hormone-related gene expression dynamics in the determination of vivipary

ABA is a well-acknowledged hormone that induces seed dormancy in the plant, and the reduction of ABA was related to vivipary (Farnsworth and Farrant, 1998). Our study illuminated the ABA reduction process with detailed temporal-spatial gene expression. The expression of the ABA synthesis genes *NCED*, *ABA2*, and *AAO3* reduced from S2 to S3, directly resulting in ABA concentration dropping drastically in the early embryogenesis period. *CYP707A* was mainly expressed in the seed coat, and the maximum being in S3 suggested no ABA accumulation in the seed coat. However, Arabidopsis seeds possess two ABA peaks; the first peak derived from maternal prevents premature seed germination, while the embryonic increase of ABA, the second peak, induces desiccation, thus the seed entering dormancy (Bradford and Nonogaki, 2007).

The high ABA concentration in Arabidopsis seeds is degraded by *CYP707A*s during the late-maturation to germination stages (Okamoto et al., 2006). Therefore, ABA metabolism gene expressions in viviparous seeds differed from orthodox seeds. There are no embryonic and maternal derived dormancy caused by ABA, and the precocious ABA reduction was a prerequisite condition set up for non-dormancy. However, GA, BR, and CK metabolism genes were more highly expressed in S3axis than S4axis, including *GA2ox, KS, KAO2*, *DWF4, CYP90A1/CPD*, *CYP735A*, and *IPT.* In the imbibed seed axis, up-regulated GA biosynthesis genes induce the subsequently cell hydrolysis in surrounding tissues (Ogawa et al., 2003). BR and GA act in parallel to counteract the inhibitory action of ABA on the seed (Steber, 2001). A peak of cytokinin content before radicle emergence was found in lettuce (Staden et al., 2010), and there is also a report showing elevated cytokinin levels by *IPT* expression can inhibit the ABA downstream genes, thus mediating Arabidopsis seed germination (Wang et al., 2011). The higher expression of these genes in the S3axis strongly suggests their positive role in stimulating axis elongation, thus promoting viviparous germination in S4.

In summary, ABA reduction and GA, BR, and CK increase in embryonic tissues in S3 is another critical change in deciding the viviparous germination.

### PAs reduction determines the non-dormancy property of the viviparous seed coat

`Seed germination is determined by the balance between the growth potential of the embryo and the mechanical restraint exerted by the surrounding tissue. Seed coat-imposed dormancy mainly originates from PAs responsible for testa thickness and mechanical strength (Debeaujon et al., 2007).

In the viviparous seed coat, PAs biosynthesis showed a reducing trend. Key PAs synthetic genes (*CHI*, *CHS*, *ANR*, *F3H*, *F3’H*, *DFR*, *LAR*, and *ANS*) drastically decreased from S2 to S3sc before germination. Mutants of the PAs biosynthesis genes are referred to as *transparent testa* (*tt*) in Arabidopsis because the testa is a lighter color. These *tt* mutants either show non-dormancy or are easier to germinate due to the less rigid testa and better permeability (Debeaujon et al., 2000). Therefore, we postulated that the reduced expression of PA biosynthesis genes was the main reason that caused the absence of seed coat-imposed dormancy and reduced constraint for germination. In comparison with an inland relative of mangrove, the non-viviparous *Carallia brachiata*, the PA metabolism in the seed was found to be dramatically different where *C. brachiata* has a dark-colored seed coat and enhanced expression of PA related gene expression (Qiao et al., 2020). Furthermore, PAs mutants also require less GA to germinate (Debeaujon and Koornneef, 2000), so even though GA biosynthesis genes are expressed lower than ABA, the viviparous embryo still germinates smoothly.

### Stress response pathways help viviparous seed inhabitation

The defense responses increase as the embryo grows. One of the most important pathways for defense is flavonoid biosynthesis. Flavonoid and phenolic compounds such as alkaloids and terpenoids are critical antioxidants in scavenging active oxygen species preventing the cell from oxidative damage in mangroves (Krishnamoorthy et al., 2011; Asha et al., 2012). These compounds also function in the reproductive organs. The embryonic axis is the only tissue that remains as propagule either plantation near the maternal tree or flotation by tidal waves. We found genes responding to UV, heat shock, osmotic stress, and DNA mismatch repair were significantly expressed in axis than in other tissues. These genes were critical in protecting the fragile embryo from intense solar radiation, high temperature, and cyclical water logging in the coastal environment (Kathiresan and Bingham, 2001).

### Conclusion and perspective

**Figure 6:**
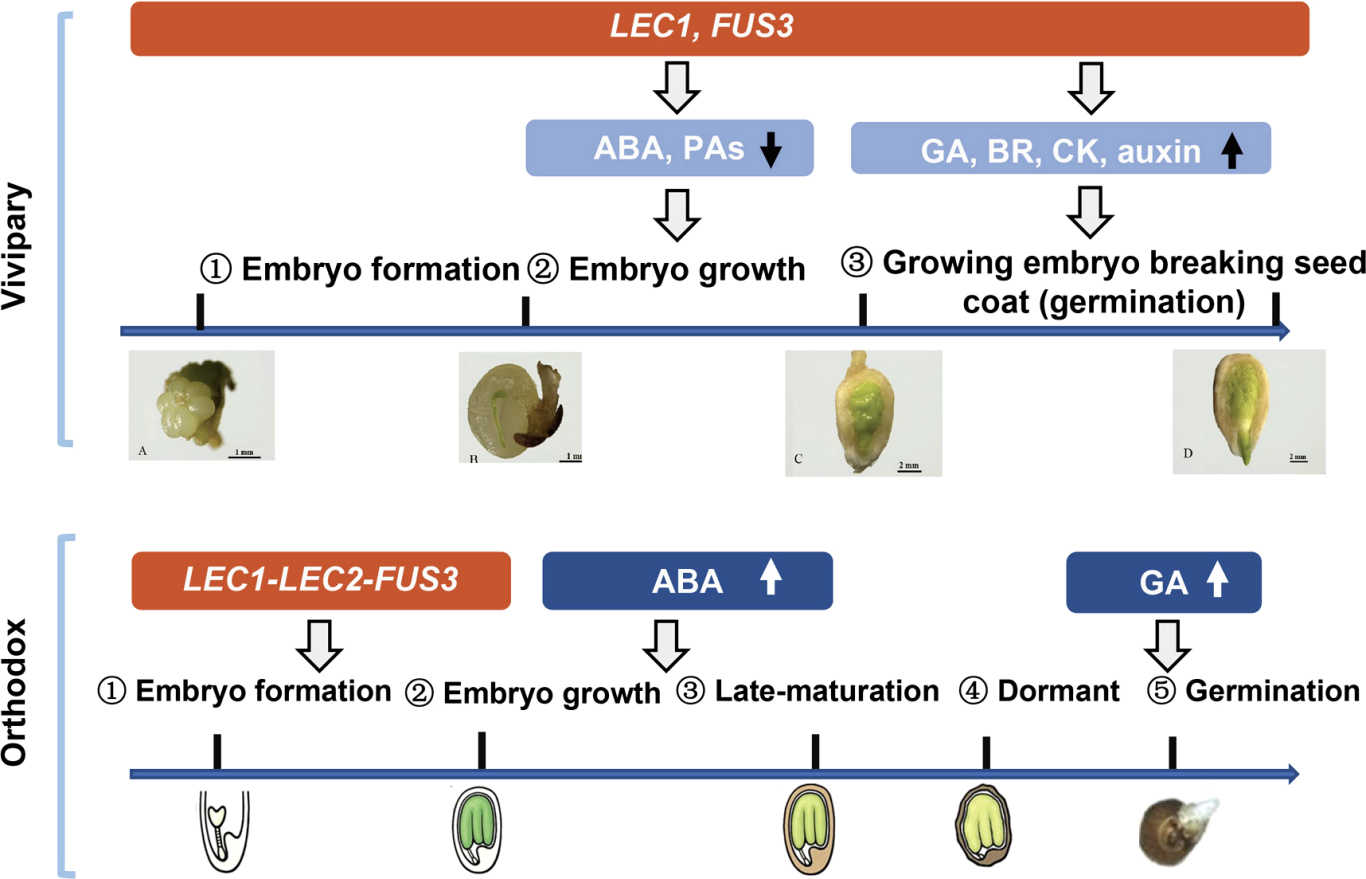
A schematic model compares the vivipary process to orthodox seed development and germination. The expression periods of key genes (*Lec1*, *Lec2*, and *FUS3*) are shaded in red. Blue shaded area hormonal and proanthocyanidin (PA) metabolic pathways, and the arrows indicate up- or down-regulation. In vivipary species like *K. obovata*, only three stages up to hypocotyl breaking the seed coat (signifying germination) are shown; after that, the hypocotyl continues to grow, breaking the fruit coat, and elongates up to 20–30 cm. Scale bars are shown in the pictures. The representative orthodox seeds are from Arabidopsis, which is not drawn in scale.

We conclude that mangrove vivipary is a coupling effect of embryo growth accompanying seed germination that is different from orthodox seeds (**Figure 6**). *LEC1* and *FUS3* contribute to maintaining the “embryo growth/embryogenesis” state that prevents the embryo from entering maturation by regulating the downstream hormone genes. Dormancy loss was caused by a decrease in ABA and PA pathway gene expression in the embryo and the seed coat from S2 to S3. Increased germination capacity induced by GA, BR, CK and auxin provided the final push in the transition from non-vivipary to vivipary. The viviparous embryo was maintained in a growth state even after germination. While in orthodox seed, the expression of *FUS3*, *LEC1*, and *LEC2* controls the embryo state and shuts down after embryogenesis; ABA and GA increase in maturation and germination stages separately.

Our results point to the direction to resolve the longstanding mystery of the vivipary mechanism, answering how vivipary occurs in mangroves and extending this new finding to broader seed biology. Future experiments are needed to explore what target proteins bind to *LEC1* to regulate embryogenesis and germination *in vivo*. This knowledge will greatly help us to reveal the role of *LEC1* in vivipary. Another open and more complex question is whether or how environmental factors, such as temperature, light, and humidity, affect vivipary occurrence.

## Data availability statement

The original contributions presented in the study are publicly available. This data can be found here: NCBI SRA, SRP261558.

## Author contributions

QQL conceived and supervised the project. XZ carried out most experiments and data analysis. YW, WS, CY, and HQ participate in data acquisition and analysis. XZ, QQL, and YW wrote the manuscript. All authors contributed to the article and approved the submitted version.
