# Supplementary material for: Uninterrupted embryonic growth leading to viviparous propagule formation in woody mangrove
Source: Front Plant Sci. 2023 Jan 4;13:1061747. doi: 10.3389/fpls.2022.1061747 (PMC9846782; doi:10.3389/fpls.2022.1061747)
Supplement: Supplementary file 1 [file Image_1.pdf]

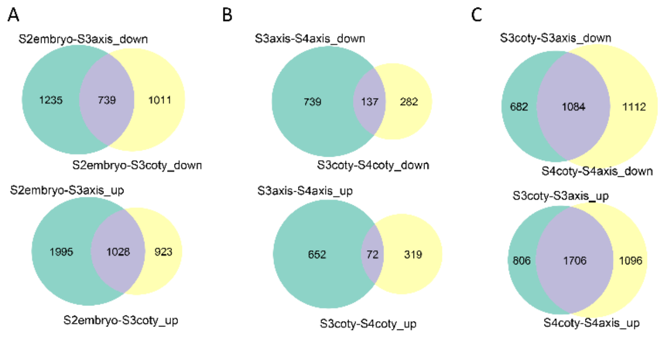

Figure S1. Venn diagrams of DEGs in different tissue comparisons.

(A) Commonly down- and up-regulated genes when comparing S2embryo to S3embryo, including axis and cotyledon, showing 739 genes expressed higher in S3 and 1028 in S2.

(B) Overlapped DEGs in the comparison of S3embryo and S4embryo in both axis and cotyledon showing 137 genes higher in S4 and 72 in S3.

(C) Duplicated DEGs of cotyledon and axis showing 1084 genes expressed higher in axis and 1706 in cotyledon.

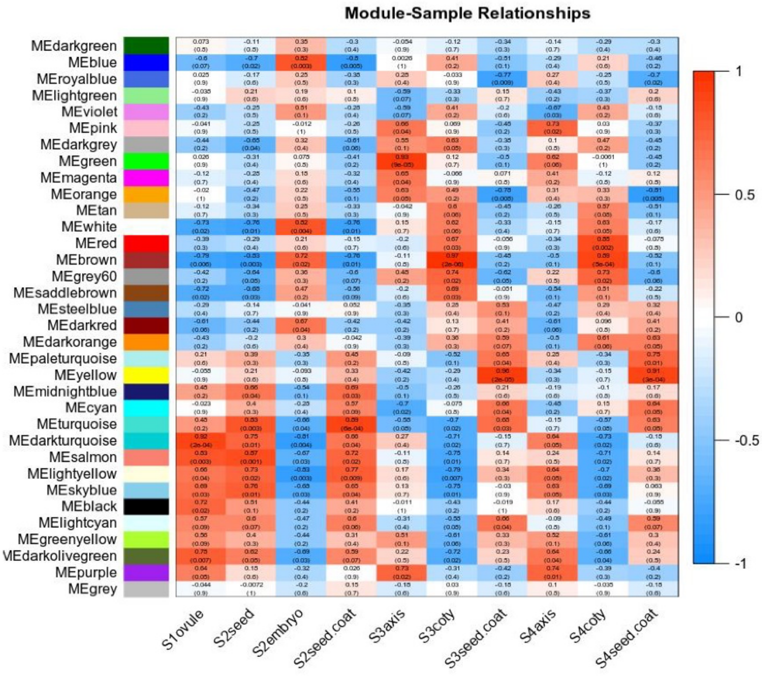

Figure S2. Module-Sample matrix (Eigengene Expression) by WGCNA analysis of viviparous tissues.

The left panel shows the cluster module names and their associated colors, the tissue names are on the bottom; boxes show the correlation index and p-value of each module; the right panel shows the scale of correlation index, the redder color means stronger relationship, while bluer means less relevance.

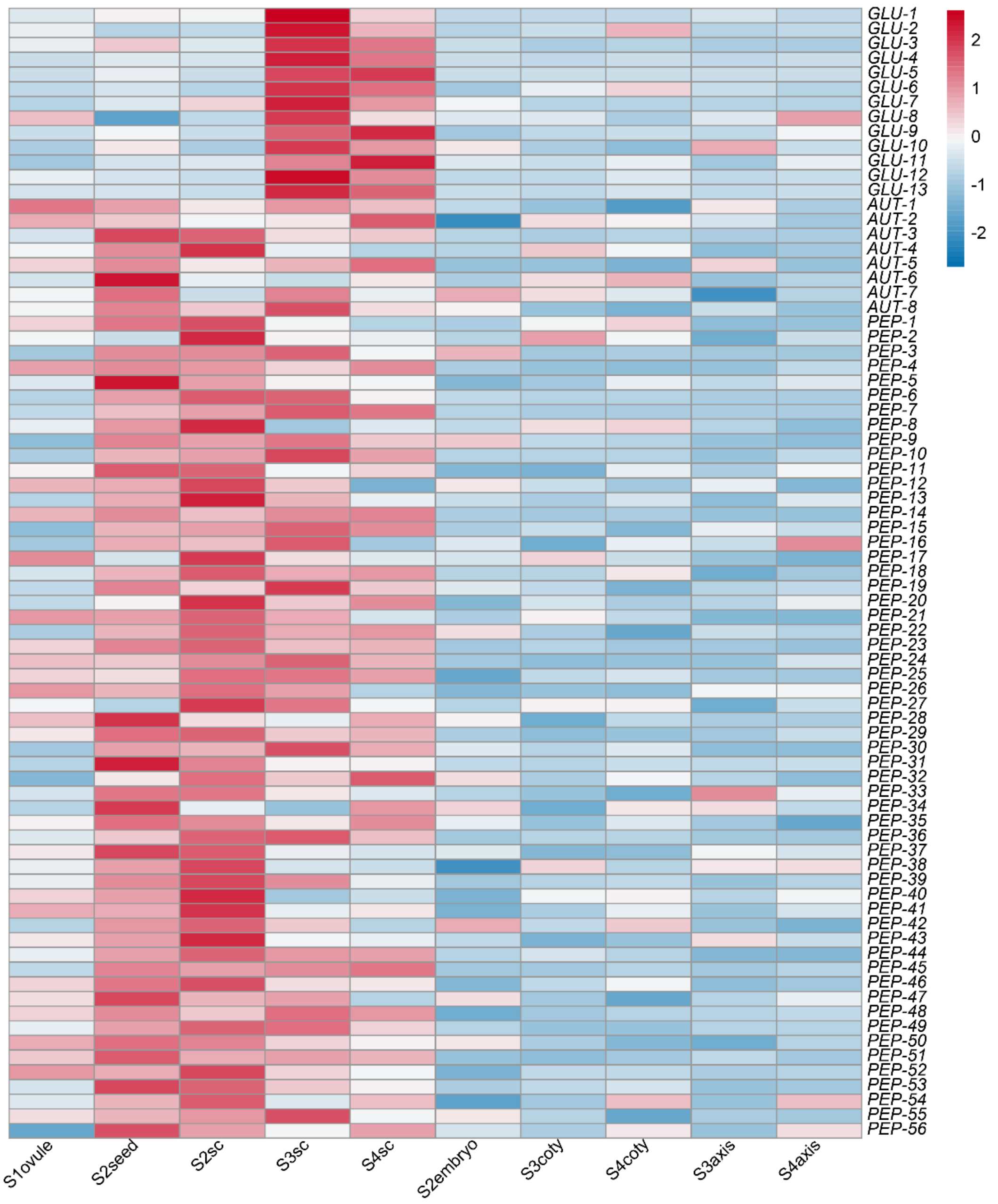

Figure S3. Clustered expression of genes function in autophagy, peptidase, glucosyltransferase.

Bars indicate z-scores, higher number and redder color means gene express more exceed the average level. GLU, glucosyltransferase; AUT, autophagy-related protein; PEP, peptidase. The scale bar is shown on the right.

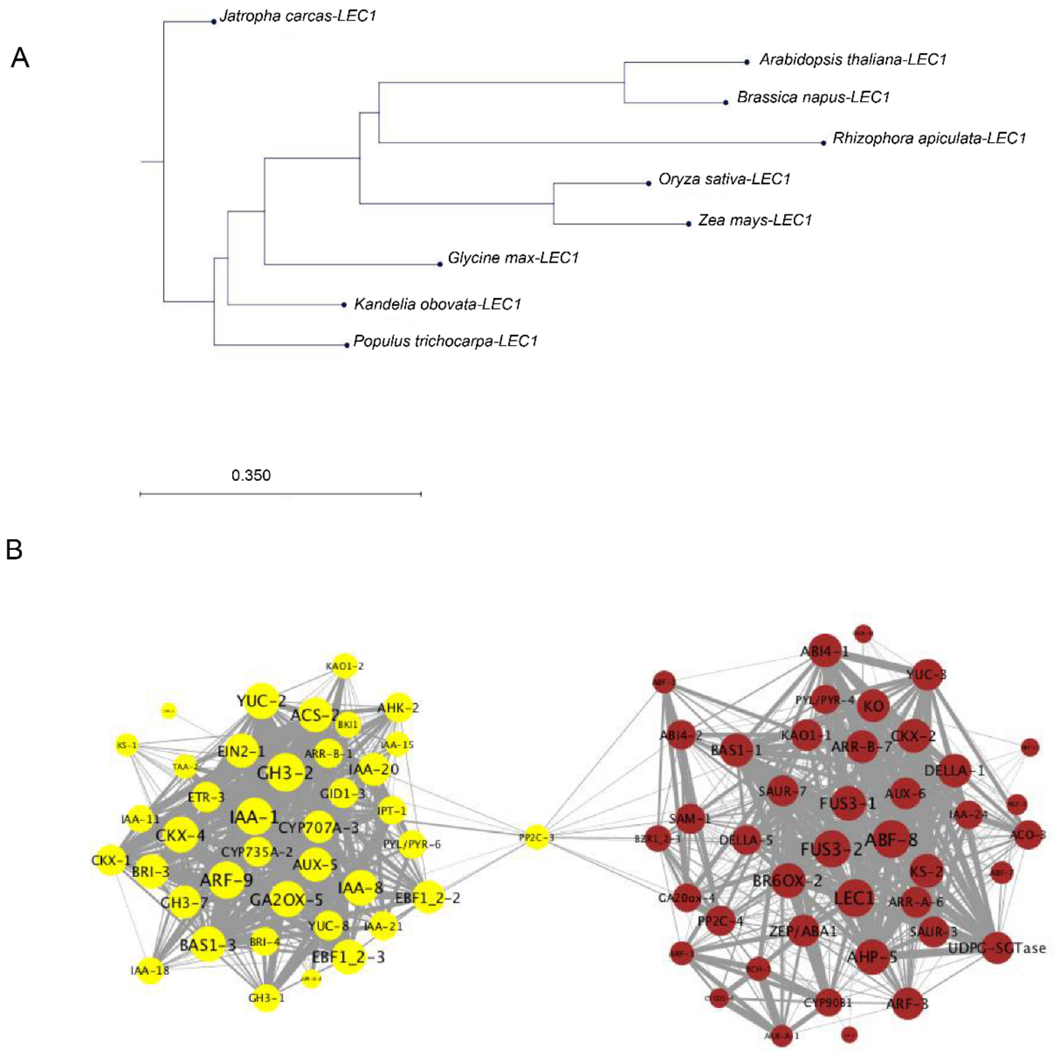

Figure S4. Phylogeny of *LEC1* and representative module connections.

(A) Phylogenetic tree of *LEC1* in 9 species constructed by maximum likehoods method showing *K. obovata* is closer to *Populus trichocarpa*, while another viviparous mangrove, *Rhizophora apiculate* is more similar to *Arabidopsis*. (B) Crosstalk between yellow and brown modules connected by *PP2C-3* with *BZR1_2-3*, *PP2C-4*, *SAM-1*, *ABF-3*, *KAO1-1*, *GA20ox-4*, *ABI4-2*, *FUS3-2*, *SAUR-7* in the brown module and *ETR-3*, *IAA-21*, *IAA-20*, *EBF1_2-2*, *GH3-2*, *BAS1-3* in the yellow module.
