# Supplementary material for: Uninterrupted embryonic growth leading to viviparous propagule formation in woody mangrove
Source: Front Plant Sci. 2023 Jan 4;13:1061747. doi: 10.3389/fpls.2022.1061747 (PMC9846782; doi:10.3389/fpls.2022.1061747)
Supplement: Supplementary file 3 [file Table_2.pdf]

Table S1. qRT-PCR primers of *LEC1*, *FUS3* and *ABI3*.

Gene ID	Gene name	Forward primers (5'---3')	Reverse primers (5'---3')
evm.model.Contig8624.509	*LEC1*	GCCAAGGAAACAATCCAAGA	CACCAGTGGCTCACTTCTCA
evm.model.Contig14326.808	*FUS3-2*	GAAAAGGATGCCCAGACAGA	GTCCGGGTCATGGAGAGTAA
evm.model.Contig5252.872	*ABI3*	TCCGCAGGCAGATTATCGCA	AGGTAGGCGAGGGATGGAGT
	ACT2	ACCGAGGCTCCTCTTAATCC	AGCTGGCACATTGAAGGTCT

Table S2. Data statistics of sequenced transcriptomes

Sample	Raw-data(GB)	Clean reads after filtered	Mapping rate to genome
S1ovule-1	5.87	13628503	89.82%
S1ovule-2	6.32	12815590	83.29%
S1ovule-3	3.61	6606472	83.54%
S2seed-1	3.1	7340100	89.41%
S2seed-2	4.24	9829676	88.55%
S2seed-3	3.71	9183111	87.88%
S2embryo-1	5.48	4919439	80.61%
S2embryo-2	8.93	4880147	78.53%
S2embryo-3	5.31	3005333	78.35%
S2sc-1	5.47	14066503	92.33%
S2sc-2	6.68	17800801	93.03%
S2sc-3	6.53	13684481	92.81%
S3aixs-1	7.14	11270817	88.63%
S3aixs-2	9.72	10447835	84.51%
S3aixs-3	8.55	13175017	89.43%
S3coty-1	7.87	13554444	84.64%
S3coty-2	7.84	14148518	83.81%
S3coty-3	6.69	11710775	87.34%
S3sc-1	7.66	25589810	93.37%
S3sc-2	7.43	24273910	92.38%
S3sc-3	7.78	26274259	91.87%
S4aixs-1	8.19	11647919	85.10%
S4aixs-2	6.54	7080519	87.45%
S4aixs-3	5.69	6955372	87.39%
S4coty-1	6.21	9807214	85.13%
S4coty-2	6.8	11390676	87.63%
S4coty-3	6.42	11065382	87.74%
S4sc-1	6.37	15040520	92.48%
S4sc-2	5.62	12165576	91.72%
S4sc-3	5.73	13131173	92.37%
Single End	193.5	366489892	
Paired End	387	732,979,784	

Table S6. Co-expressed genes that are commonly down regulated by *LEC1-FUS3-ABI3* in WGCNA.

Gene name	ID	Pathway	Total weigh
ZEP/ABA1	evm.model.Contig9868.281	ABA biosynthesis	0.33
ABA2-4	evm.model.Contig14326.505	ABA biosynthesis	0.29
SAUR-7	evm.model.Contig8624.114	auxin response	0.29
CYP90B1	evm.model.Contig5386.245	BR biosynthesis	0.27
BCH-3	evm.model.Contig2712.1346	ABA biosynthesis	0.27
ARR-A-1	evm.model.Contig8573.231	cytokinine response	0.23
ARF-3	evm.model.Contig2800.462	auxin response	0.2
PP2C-4	evm.model.Contig2581.703	ABA response	0.2
ARF-1	evm.model.Contig5386.879	auxin response	0.2
GA20ox-4	evm.model.Contig2712.1242	GA biosynthesis	0.17
BZR1_2-3	evm.model.Contig8624.46	BR response	0.16
